# Sarcopenia: A Major Challenge in Elderly Patients with End-Stage Renal Disease

**DOI:** 10.1155/2012/754739

**Published:** 2012-03-28

**Authors:** Maciej Domański, Kazimierz Ciechanowski

**Affiliations:** Department of Nephrology, Transplantation and Internal Medicine, Pomeranian Medical University, 70-111 Szczecin, Poland

## Abstract

Sarcopenia is a condition of multifaceted etiology arising in many elderly people. In patients with chronic kidney, the loss of muscle mass is much more intensive and the first signs of sarcopenia are observed in younger patients than it is expected. It is associated with the whole-body protein-energy deficiency called protein-energy wasting (PEW). It seems to be one of the major factors limiting patient's autonomy as well as decreasing the quality of life. If it cannot be treated with the simple methods requiring some knowledge and devotion, we will fail to save patients who die due to cardiovascular disease and infection, despite proper conduction of renal replacement therapy. Many factors influencing the risk of sarcopenia development have been evaluated in number of studies. Many studies also were conducted to assess the efficacy of different therapeutic strategies (diet, physical activity, hormones). Nevertheless, there is still no consensus on treatment the patients with PEW. Therefore, in the paper we present the reasons and pathophysiology of sarcopenia as an important element of protein energy wasting (PEW) in elderly patients suffering from chronic kidney disease. We also analyze possible options for treatment according to up-to-date knowledge.

## 1. Introduction

The development of dialysis and the increasing availability of this renal replacement therapy method is the reason elderly patients have become a growing population in dialysis centers. The average age of hemodialyzed patients in many countries exceeds 60 years. Sarcopenia is a condition arising in many elderly people mainly as a result of reduced physical activity, although a lot of data suggests a multifaceted etiology of the phenomenon. An adult without chronic kidney disease should expect a loss of muscle mass reaching 1% per year [1, 2]. Very often it can be reduced through appropriate physical training [3].

Patients with chronic kidney disease experience probably the same age-related processes. It is interesting, however, that in those patients the loss of muscle mass is much more intensive. Moreover, the first signs of sarcopenia in CKD patients are observed earlier compared to their peers. It is very well marked in patients with the end-stage renal disease (ESRD or CKD V according to K/DOQI guidelines), who are treated with various methods of renal replacement therapy. The most significant aberrations have been recorded in patients treated with hemodialysis.

The question is, what factors can influence such situation and whether sarcopenia developing in healthy people over 60 years old has the same pathogenic basis as the one developing in CKD patients.

Many authors have studied the phenomenon of muscle wasting. According to the International Society of Renal Nutrition and Metabolism (ISRNM), it is a significant element of the condition defined as protein-energy wasting (PEW). It describes the organism's state of reduced protein and energy resources. It is assumed that 18–75% of patients with CKD suffers from PEW [4–6].

According to the ISRNM statement, there are 4 main criteria useful in PEW diagnosis:

1. low body weight, reduced body fat and weight loss,
2. reduced muscle mass judged on the basis of mid-arm circumference, dual X-ray absorptiometry, near-infrared interactance, bioelectrical impedance, or creatinine appearance,
3. low protein/energy intake,
4. biochemical indicators [7].

BMI is one of the methods to assess low body weight, although in patients with ESRD it might be inappropriate, because of fluid imbalance [8, 9]. Some of the authors suggest subjective global assessment (SGA), as a precise marker of body weight loss. Undisputedly, it is more complicated, as it requires not only a simple mathematic operation but is based on medical history and physical examination. Stenvinkel et al. proved that it might be useful in assessment of ESRD patient outcome as SGA ≥ 2 is correlated significantly with increased cardiovascular mortality [10]. 

Unintentional weight loss of at least 5% of body weight in 3 months or ≥10% in 6 months is another (independent of BMI) indicator of PEW. In patients with CKD, body fat mass less than 10% may also be pathognomonic for PEW [11]. 

Reduced muscle mass seems to be the most important element of PEW. Mid-arm circumference is a simple, but precise method of muscle loss assessment. Noori et al. proved it is correlated with the survival and quality of life in patients on hemodialysis [12]. All other—mentioned above—methods are not widely used, because of their limitations (particularly concerning water imbalance) in ESRD [13]. Only creatinine appearance (assessed by quantification of creatinine in 24 h urine collection and in the collected spent dialysate) is useful, but labor-consuming method [14]. 

Both low protein/energy intake and anorexia are associated with high cardiovascular risk. In fact, anorexia is a severe form of low protein/energy intake, very often connected with cachexia. In many cases, it is associated with high concentrations of proinflammatory factors and ESA resistance leading to low quality of life and increased mortality [15]. Unintentional dietary energy intake (DEI) lower than 25 kcal/kg/day for at least 2 months or unintentional dietary protein intake (DPI) lower than 0.8 g/kg/day for at least 2 months are criteria for low protein/energy intake [7].

Serum albumin is the most important biochemical indicator of PEW in patients on maintenance dialysis. It should be controlled regularly, as serum albumin still remains the most readily available PEW indicator with very high sensitivity of predicting ESRD patient outcome. The difference in serum albumin as little as 20 mg/L may be crucial. Low serum albumin concentration is the strongest predictor of mortality in ESRD patients, even when compared to traditional cardiovascular risk factors (obesity, diabetes, smoking, hypertension, dyslipidemia) [16–19]. 

Low serum prealbumin concentration is another good PEW indicator in patients on maintenance dialysis. It was proved, that the concentration below 20 mg/dL or a fall in serum prealbumin over 6 months is an independent risk factor for death, even when the albumin level remains in normal range [20, 21]. Rambod et al. have made an interesting finding according prealbumin, which might turn into an option, when it comes to sarcopenia treatment. They found the positive correlation between serum prealbumin concentration and percentage muscle mass, as well as inverse association between serum prealbumin and percentage body fat [21].

Transferrin serum level as well as serum cholesterol (<100 mg/dL) may also serve as protein energy wasting indicators [22, 23]. 

The interest in the phenomenon of protein and energy loss seems to be obvious when we realize what important consequences result therefrom. First of all, it is one of the main predictors of mortality in patients with ESRD [1, 10, 24]. It also causes a significant reduction in quality of life. Loss of lean body mass may lead to frequent falls, osteoporosis, and its complications, thus limits the autonomy of patients and may be a cause of disability [25]. Besides the protein degradation, the accompanying fat accumulation in the visceral area favors the development of insulin resistance and, consequently, the metabolic syndrome and many of its consequences [25].

### 1.1. What Happens to the Protein and to the Skeletal Muscles in ESRD Patients?

Many authors have evaluated the degradation of skeletal muscle protein in their studies trying to link the observed phenomena with the metabolic state of the organism. During hemodialysis, we observe enhanced proteolysis in muscle. It should be noted that about 10 g of amino acids are irreversibly lost to the dialysate during hemodialysis [26]. It may cause a loss of muscle mass exceeding 1% per year. Amino acids infusion though in the study of Raj did not bring expected results of muscle regeneration [26]. 

An unrecognized steering mechanism causes the products of muscle protein breakdown which are released to the blood and transported to the liver, where they serve probably as substrates for acute phase proteins synthesis [26–28]. The hypothesis could explain the inflammatory system activation in CKD patients.

Not only the protein loss and not just reduction of muscle mass affect the patients' functioning, however. Another negative phenomenon is the change of the quality of the muscular compartment. In patients with ESRD, we observe both the accumulation of fat in the striated muscle cells (intramuscular lipid) and in the form of intermuscular adipose tissue (IMAT), subfascially [1]. 

In the study with 49 hemodialyzed patients, Cheema et al. demonstrated that such disturbed muscle structure is more common in older people, with hypoalbuminaemia and high levels of proinflammatory cytokines. IMAT was associated particularly with high BMI and larger waist circumference. The researchers pointed out that this influenced the functioning of patients with statistically significant reduction of physical fitness, which was confirmed by objective methods (reduced gait velocity, decreased 6-minute walking distance, peak isometric strength) [1]. Honda et al. examining obese (BMI > 30) patients with ESRD found a severe expression of inflammation markers, which was probably derived from the adipose tissue [29].

## 2. The Mechanisms of the Sarcopenia Development in Elderly Patients with Chronic Kidney Disease

Global dialysis development brought us the possibility of observation made in many patients with ESRD, including of course, the anatomical and functional assessment of the muscular system. Many reports have described the mechanisms of muscle tissue destruction.

**Figure 1:**
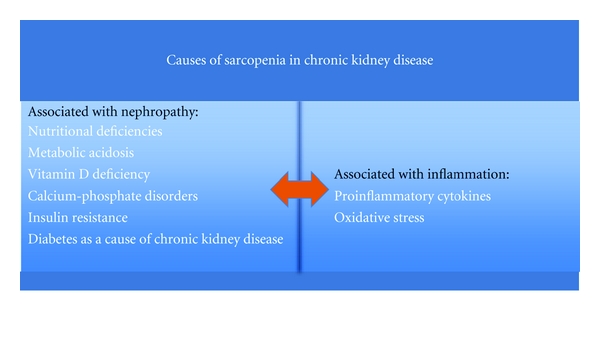
Causes of sarcopenia in chronic kidney disease patients.

The current state of knowledge indicates that the factors predisposing patients with chronic kidney disease to the development of sarcopenia in fact can be divided into two groups (Figure 1):

1. associated with the kidney disease (nutritional deficiencies, development of acidosis, vitamin D deficiency and calcium-phosphate disorders, insulin resistance, diabetes as a cause of chronic kidney disease, sometimes proteinuria),
2. associated with the developing inflammatory process, particularly characteristic for hemodialysis patients, but also observed in patients not enrolled in the chronic dialysis [26, 29].

### 2.1. Factors Associated with Chronic Kidney Disease Contributing to the Development of Sarcopenia

Nutritional deficiencies seem to be the most important reason of muscle loss in ESRD patients. Besides socioeconomical causes (very significant in many countries) leading to malnutrition, there are some other bases for inadequate protein/energy intake. Altered taste, gastroesophageal reflux, and impaired gastric emptying (particularly in patients with diabetic gastroparesis) are the most common. The phenomenon is potentiated by depression development, quite frequent in patients with chronic diseases. It leads not only to low protein/energy intake, but also may promote proinflammatory status. Bellisle et al. and Hung et al. found the association between depression and high IL-6 serum concentration, which was correlated with low serum albumin level [30, 31]. 

In patients with ESRD, inadequate nutrition is also a consequence of leptin and ghrelin perturbation. In a healthy human, kidney is the place of the two hormones degradation. With decreasing GFR, the metabolism of ghrelin and leptin becomes insufficient. Increased concentration of proinflammatory cytokines also contributes to leptin accumulation [32, 33]. 

The data concerning leptin influence on PEW remain conflicting, although some experimental data bring some hope for future treatment. Cheung et al. in their study proved that leptin signaling through hypothalamic melanocortin-4 receptor blockade leads to cachexia amelioration in mice. The authors pointed, that it could be only one of many pathways, as some indicators of excessive muscle degradation remained high during the study [34]. 

The role of ghrelin in ESRD-PEW development remains uncertain. There are two major forms of circulating ghrelin: acylated ghrelin and des-acyl ghrelin. The latter is responsible for negative energy balance and that form is probably associated with low protein/energy intake [35]. Nevertheless, the role of ghrelin in PEW development requires further investigation.

Metabolic acidosis inevitably accompanying chronic kidney disease, especially in its advanced stages (when it comes to glomerular filtration rate reduction below 25 mL/min/1.73 m^2^), is one of the most common reasons of PEW [36–38]. Its multifaceted impact on muscle in patients with chronic kidney disease is via impairment of albumin synthesis, increased protein and branched-chain amino acids degradation, and increased proinflammatory cytokine synthesis. [38]. It is been postulated recently, that the main reason for sarcopenia secondary to metabolic acidosis is increasing insulin resistance [39]. Oral bicarbonate therapy, as it is advised in patients with GFR below 30 /mL/min/1.73 m^2^ might reduce the impact of these metabolic disorders [38]. It proved to be efficient in insulin resistance improvement, probably by enhanced synthesis of 1,25(OH)_2_D_3_ [40].

Insulin resistance has appeared as one of the most important metabolic challenges in patients with CKD [41]. Diabetes is an independent risk factor for muscle wasting in patients with ESRD [26, 42]. Pupim et al. proved that in patients with ESRD, the loss in lean body mass was significantly increased in those who suffered from diabetes [42, 43]. The possible mechanisms are the concomitant insulin resistance, the intensity of inflammatory processes, and, sometimes, massive loss of protein in the urine [44]. Type 2 diabetes is the most common cause of chronic kidney disease observed in hemodialysis patients. Therefore, it seems that reducing the impact of accompanying metabolic disorders on the muscle may significantly result in survival improvement and better quality of life in the dialysis patient population.

Insulin resistance is observed also in nondiabetic and nonobese patients on maintenance dialysis. It is associated with increased muscle protein breakdown mainly mediated by ubiquitin-proteasome pathway (described below) [45]. It was also proved that insulin resistance leads to decrease in muscle PI3K (phosphatidylinositol 3 kinase), which is the reason for high ubiquitin-proteasome system activity, but also a reason for Bax-dependent caspase-3 hyperactivity leading to enhanced protein degradation. Some data suggest that peroxisome proliferator-activated receptor (PPAR) agonists (thiazolidinediones) may reverse this mechanism [46, 47].

The more effective treatment of secondary hyperparathyroidism in chronic kidney disease is probably the reason the calcium-phosphate metabolism disturbances are not the main problem in the development of sarcopenia. There is still a phenomenon of muscle weakness (mainly proximal muscle groups) in patients with uncontrolled hyperparathyroidism. Moreover, some authors treat myopathy as an indication for parathyroidectomy [48, 49]. Now it seems that the optimization of dialysis, the appropriate pharmacological action, in the absence of parathyroid adenoma (which should always be ruled out), may help to prevent muscular complications.

Vitamin D deficiency associated with renal failure also contributes to the development of myopathy, mainly through increasing insulin resistance [50, 51]. In 1980, Norman et al. showed that vitamin D deficiency affects insulin secretion by pancreatic islet *β*-cells [52], and few years later they proved beneficial effects of vitamin D supply on glucose tolerance and decreased insulin resistance [53]. This was confirmed also in chronic kidney disease [54].

In some patients, proteinuria in the course of primary kidney disease may lead to significant protein losses. It may be an important reason in patients with earlier stages of chronic kidney disease. During hemodialysis, with decreasing residual diuresis, this way of protein loss becomes less important.

### 2.2. Chronic Kidney Disease and Activation of Inflammatory Reaction

The fact that chronic kidney disease is a state of permanent inflammatory process has been known for many years [1, 26, 55, 56]. Cytokine signaling through nuclear factor kappaB (NF*κ*B) results in a stimulation of enzymatic systems (described below) favoring muscle proteolysis [26, 55]. TNF*α*, IL-6, IL-8, and IFN-*γ* are mentioned among the most frequently observed indicators and stimulators of muscle proteolysis, while C-reactive protein (CRP) seems to be a useful and inexpensive, although nonspecific, marker of systemic inflammation [4, 57]. Metabolic acidosis, hormonal disorders (mainly related to the accumulation of adipose tissue hormones—leptin and adiponectin, and ghrelin deficiency), and blood contact with synthetic dialysis membranes in hemodialysis patients are the known factors increasing the synthesis of proinflammatory cytokines [57–59]. The main sources of inflammatory cytokines are activated lymphocytes and macrophages, adipose tissue, and the skeletal muscles themselves [26]. It is assumed that persistent inflammation causes abnormal utilization of amino acids administered during hemodialysis. They are not built into the muscles, as we would expect during muscle reconstruction after proteolysis. Amino acids are transported to the liver where they are utilized probably to the synthesis of acute phase proteins [7, 60]. Experimental attempts to block TNF*α* and NF*κ*B inhibited skeletal muscle proteolysis [61, 62].

Permanent inflammation promotes oxidative stress, and thus the formation of oxygen free radicals. That is another factor exacerbating inflammatory response by affecting NF*κ*B especially with impaired mitochondrial function and reduced antioxidant capacity. It is obvious that inflammatory activation and oxidative stress are not only harmful to the kidneys of patients with chronic kidney disease, but also affect the cardiovascular system, therefore, contribute to the premature death of patients with chronic kidney disease [4, 44]. 

Chronic inflammation promotes reduced muscle mass by inhibiting insulin signaling and increasing energy expenditure, which cannot be covered by appropriate energy intake in ESRD patients. Increase energy expenditure in terms of inadequate energy intake is a characteristic feature of PEW. Besides activation of proinflammatory processes, also increased activity of mitochondrial anion transporters-uncoupling proteins (UCP) may lead to increased resting metabolic rate. UCP-1 and UCP-3 seem to be key regulators of energy expenditure in humans [63–65]. Cheung et al. proved both proteins are expressed intensively in uremic mice [34]. Uncoupling of mitochondrial electron transporter chain activity from the ADP phosphorylation leads to enhanced thermogenesis and subsequently high-energy expenditure [64].

### 2.3. Molecular Mechanisms of Muscle Proteolysis in Chronic Kidney Disease

Three main systems are involved in the degradation of muscle proteins: the cytosolic calcium dependent calpain system, the lysosomal proteases (cathepsin system), and ubiquitin-proteasome system (UPS) [26, 58]. The available studies show the first two systems are not very active in terms of increased catabolism, and their inhibition does not contribute to proteolysis suppression [58, 66]. Therefore, the UPS remains the major pathway of skeletal muscle protein degradation [67].

**Figure 2:**
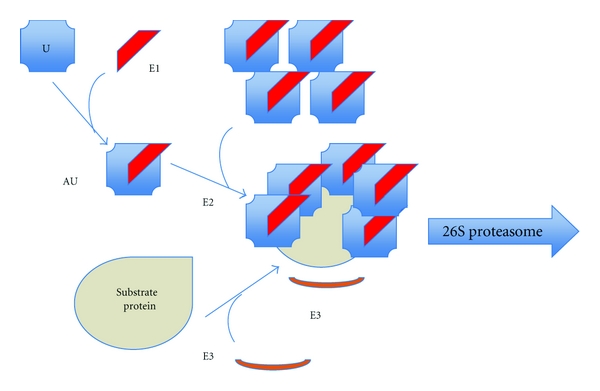
Ubiquitin-proteasome system. U: ubiquitin, AU: activated ubiquitin, E1: ubiquitin-activating enzyme, E2: ubiquitin-conjugating enzyme, E3: ubiquitin ligase.

Ubiquitin is a member of the heat shock protein family. It is activated by the ubiquitin-activating enzyme (E1), which is followed by its transfer to an ubiquitin-conjugating enzyme (E2). E2 transfers the activated ubiquitin moieties to the protein substrate that is bound specifically to a unique ubiquitin ligase E3. Successive conjugation of ubiquitin moieties to one another generates a polyubiquitin chain (containing 5 ubiquitin moieties) that serves as the binding signal for the 26S proteasome degrading the target substrates to peptides [41, 58] (Figure 2). Peptides are than hydrolyzed to amino acids by peptidases in cytoplasm. 

Impaired insulin/IGF-I signaling is another pathway leading to muscle degradation. The lack of IGF-I receptor stimulation is most commonly caused by metabolic acidosis, elevated angiotensin II, and chronic inflammation. Inhibiting of Act phosphorylation leads to Caspase-3 activation and thus to severe proteolysis. The reduction of a forkhead transcription factor (FOXO) phosphorylation is another effect of impaired IGF-I receptor stimulation. In such conditions, FOXO is transited to the cell nucleus and stimulates the synthesis atrogin-1/muscle atrophy f-box (MAFbx) and Muscle ring finger 1 protein (MuRF1). They belong to a group of UPS E3 enzymes that recognize specific muscle proteins, leading to their degradation [58, 68]. An intensive MuRF1 expression is also the reason for hypercortisolaemia-induced myopathy [69]. 

Myostatin is another protein having its negative impact on the development of sarcopenia in patients with chronic kidney disease. Its role is to block the expression of MyoD that enhances myogenesis. Sun et al. have demonstrated increased expression of myostatin in the muscle of rats with end-stage renal disease. They also indicate at the imbalance between myostatin and IGF-I as one of the mechanisms responsible for muscle atrophy in chronic kidney disease [70].

## 3. Methods of Sarcopenia Prevention and Treatment in Elderly Patients with Chronic Kidney Disease—Could It Be Effective?

Based on the above-cited advances in understanding the mechanisms causing sarcopenia, several studies were designed to propose some treatment and improve patients' survival and their quality of life.

### 3.1. Physical Activity and Nutrition

It seems that the implementation of appropriate training in patients with chronic kidney disease both in predialysis period as well as during dialysis can reduce the risk of sarcopenia. This action also modulates the risk of cardiovascular diseases—the leading cause of death in patients with ESRD. Therefore, it is a strongly recommended strategy to implement in CKD patients both young and elderly. Storer et al. proved that endurance exercise might improve strength, power, and physical performance in haemodialysis patients [71]. Probable mechanisms include improved insulin sensitivity, decreased synthesis of pro-inflammatory cytokines, improved conduction through the IGF-I, and thus also UPS pathway depression. Storer et al. have confirmed the hypothesis of the inflammatory response inhibition in the studies, where the CRP and IL-6 concentrations were diminished as a result of twelve-week resistance training in patients on dialysis [71]. Smart and Steele observed a reduction of TNF*α* synthesis in their meta-analysis concerning patients with chronic heart failure, in whom physical therapies employing ≥5 sessions per week were introduced [72].

Cheema et al. studied an impact of 12-week progressive resistance training (PRT) administered during hemodialysis on skeletal muscle quantity and quality. They showed that PRT is not sufficient in LBM improvement but proved a statistically significant improvement in muscle strength, attenuation, mid-arm, and mid-thigh circumference comparing to group treated in a usual way [73].

Johansen et al. in their study with anabolic steroid administration and resistance training proved there was an increase in quadriceps cross-sectional area. Unfortunately, they did not notice any increase in lean body mass in the group of patients, where exercise was the only therapeutical method. Nevertheless, their study showed some improvement in self-reported physical functioning [74]. The study by Kopple et al. revealed a slight increase in lean body mass assessed by dual-energy X-ray absorptiometry. It was correlated with upregulation of mRNA and protein of IGF-I in skeletal muscles [75].

According to our best knowledge in 29 clinical trials, the aerobic training improved patients' physical condition (by increasing exercise capacity and exercise duration) [76]. Resistance training was beneficial for patient muscle strength and their functional capacity (increase in the peak torque of quadriceps muscle, improved walking-distance in a 6-min walk test, improved walking speed, and improved performance in a sit-to-stand test) [77].

In a number of studies, the beneficial effects of both aerobic and resistance training were proved. Deligiannis et al. showed that aerobic physical training leads to improved cardiac function measured by ejection fraction, cardiac output index, and systolic volume index [78]. Kouidi described also an increase in cardiac vagal activity, diminished sympathetic hyperactivity, and less incidence of arrhythmias in CKD patients treated with appropriate physical activity [79].

Physical activity also seems to have an influence on arterial hypertension control both—during hemodialysis and in the interdialytic period [80–82]. It is important to continue the treatment, as Boyce et al. noticed, that after 2 months of exercise cessation high values of systolic and diastolic treatment returned [83], probably due to increasing arteries stiffness [84].

In terms of metabolic changes caused by physical activity, decreased concentration of VLDL and triglycerides combined with improved level of HDL was proved by Goldberg et al. [85] The knowledge of improved insulin resistance in physically active patients is common and has been confirmed in the number of trials [86].

Parsons et al. and Oh-Park et al. proved that adequate physical activity may improve hemodialysis efficiency. The probable reason for such situation is increased muscle perfusion, which occurs during exercise. It leads to the transfer of uremic toxins concentrated in muscles to the circulation; therefore, they can be easily removed during hemodialysis. A so-called “post-dialysis rebound” (increased urea, creatinine, potassium after hemodialysis due to the slow transfer from poorly perfused regions, i.e., skeletal muscles) is diminished in physically active patients [87–89]. 

Some authors concentrated their efforts on proving a beneficial effect of exercise during hemodialysis on skeletal muscle fibers. A resistance training leads to reduction of atrophic fibers number, an increase in type I, type IIa, and type IIx muscle fibre cross-sectional areas. [90]. Also aerobic training in CKD patients may lead to such changes in muscle structure, which is not typical in general population. Probably the large potential for skeletal muscle improvement in CKD patients is the reason for this phenomenon [91].

The great value of physical exercise for improved psychological adaptation also has been researched. Kouidi et al. showed that Beck's Depression Index was decreased in patients physically active, which was confirmed in the Ouzouni et al. research [92, 93]. In the studies of Oh-Park et al. and Painter et al. the mental health and health-related quality of life (HRQoL) improvement assessed on the basis of SF-36 QoL scale was presented, although the evidence remains moderate due to lack of statistical significance [88, 94]. Cheema et al. proved a statistically significant positive impact of resistance training on HRQoL (also using SF-36 QoL scale) [73]. As depression is one of the reasons for low protein/energy intake, some authors also proved an increase in appetite (and subsequently DEI and DPI) [95]. 

As inadequate protein/energy intake and high energy expenditure are characteristic features of PEW, a proper nutrition seems to be a natural treatment option to choose in patients on maintenance dialysis. Up to date, the influence of nutritional therapy in ESRD patients was researched in 13 randomized trials. The results of the most important ones are presented in Table 1 [96–104]. Most of them were performed on small groups of patients, which make any analysis concerning sarcopenia treatment difficult. The combination of a proper nutrition with exercise, nevertheless, might be an efficient treatment option [105, 106]. Cheung et al. using a mouse model of CKD proved, however, that nutritional supplementation may contribute to wasting improvement and decreasing metabolic rate in CKD, but it will not fully correct them [34, 107]. As it was mentioned earlier, attempts to supply amino acids during dialysis did not produce the expected results [26].

The effect of appetite-stimulating agent, megestrol acetate, is still not enough documented in randomized controlled trials in large groups of the patients. In the studies designed by Boccanfuso and Burrowes on a small group of patients with ESRD, the risk of adverse events exceeded the benefits of megestrol use [108, 109]. They noticed no significant increase in LBM, but reported many side-effects instead (headache, dizziness, confusion, diarrhea, hyperglycemia, thromboembolism, peripheral edema, hypertension, and adrenal insufficiency). Some recent studies, although, have proved megestrol acetate has a beneficial effect not only according to the nutrition status, but also improving inflammatory parameters [110, 111].

### 3.2. Hormone Therapy

Some very promising data from studies with recombinant human Growth Hormone (rhGH) has been published so far. Pupim et al. observed its beneficial anabolic effect in hemodialysis patients in the short-term therapy [112]. The increase in muscle protein synthesis was proved by Garibotto et al. who administered 50 ug of rhGH for 6 weeks in patients with cachexia and ESRD [113]. Feldt-Rasmussen et al. proved beneficial effect of rhGH on LBM and quality of life in patients with ESRD. What is very important is that they did not notice any significant side effects [114]. Still more controlled trials are required to evaluate the effect of rhGH therapy both in predialysis and dialysis patients. 

Wynne et al. and Ashby et al. assessed ghrelin subcutaneous injection in patients with ESRD, but the results proved only some improvement in short-term energy intake [115, 116]. There are no trials concerning possible ghrelin administration effect on muscle metabolism.

Cheung et al. demonstrated an important role of leptin in PEW development. They also proved that the blockade of melanocortin receptor 4 (MC4-R), by AgRP or NBI-12i, attenuated uremic cachexia in mice [34, 107]. As an effect of the therapy, the mice gained lean body mass and fat mass in comparison to the control group treated only with an appriopriate diet. Their basal metabolic rate was decreased, which shows that some other studies concerning leptin and melanocortin signaling pathway are necessary to expand and enhance the treatment arsenal.

### 3.3. UPS Suppression

UPS is one of the central pathways that contribute to muscle breakdown. It seems that the current therapy proposals should be directed to modification of this system. Bortezomib—now used in cancer therapy proteasome inhibitor—can affect the UPS system by inhibiting NF*κ*B signalling [117]; however, possible implementation of such treatment requires further investigation.

### 3.4. Optimizing Hemodialysis

Hemodialysis patients are particularly vulnerable to the development of sarcopenia, mainly due to strong stimulation of the inflammatory response during hemodialysis. Attempts have been made, therefore, to shorten the exposure of blood to the dialysator membranes by reducing the time of hemodialysis, in exchange for increasing their frequency. Galland et al. observed a beneficial effect of such proceedings in 2 studies, with the increase in lean body mass, serum albumin, prealbumin, and cholesterol concentration during the 6- and 12-month followup [118, 119]. The studies, however, were conducted on small groups of patients (*n* = 8, *n* = 17), although one should appreciate the relatively long observation period. Similar observations have never been confirmed in a large patient population.

### 3.5. Treatment of Acid-Base and Calcium-Phosphate Metabolism Disorders

An active form of vitamin D3 deficiency characterizes chronic kidney disease. Its supplementation may result in a reduction of insulin resistance and reduced activity of the UPS. Mak conducted a study with only intravenous use of vitamin D3. Intravenous 1,25(OH)_2_D_3_ therapy corrected glucose intolerance, insulin resistance, and hypoinsulinemia as well as hypertriglyceridemia in patients on HD, in the absence of PTH suppression [54].

In the studies using oral vitamin D3, the results are conflicting [120]. Nevertheless, up to now there have been no big trials concerning the impact of vitamin D3 therapy on muscle mass. Therefore, the beneficial, though indirect (by insulin resistance improvement), effect of such treatment remains a hypothesis.

There is no doubt that the proper treatment of the metabolic acidosis accompanying chronic kidney disease may inhibit the development of sarcopenia [121, 122]. In patients with ESRD, an adequate dialysis leads to acidosis correction. However, oral supplementation with NaHCO_3_ proved to be effective improving nutritional status and lean body mass. The benefits are probably caused by the impact on the UPS system, reduced insulin resistance [123], and perhaps also on increased activity of 1*α*-hydroxylase in the kidneys and thereby increased synthesis of the active form of vitamin D [54]. Pickering et al. in their study proved that using a dialysate with higher lactate concentration leads to decrease in protein degradation most probably by downregulation of UPS system. An increase in branched-chain amino acids was also observed in the study. The study was conducted in small group of patients, and the observation period was only 4 weeks [123]. Though it confirmed an earlier observations made by Stein et al. of beneficial influence of acid-base balance correction in ESRD patients. In his group of 200 patients on peritoneal dialysis, the dialysate solution of 35 mmol/l or 40 mmol/l lactate contributed to higher serum bicarbonates concentration. It was followed by weight gain and increased muscle mass measured by mid-arm circumference [122].

In patients with CKD in predialysis period, oral supplementation of NaHCO_3_ was effective in increasing DPI/DEI as well as muscle mass (assessed by mid-arm circumference) and serum albumin concentration [123, 124].

## 4. Conclusion

Sarcopenia is a common problem in patients with chronic kidney disease. In those patients, a number of clinical problems have its origins much earlier than in healthy peers. Sarcopenia also evolves much earlier than in the population without chronic kidney disease, and its development is much more rapid. It is associated with the whole-body protein-energy deficiency called protein-energy wasting (PEW).

Sarcopenia, as an important element of PEW, is the factor limiting patient's autonomy as well as decreasing the quality of life. If it cannot be treated with the simple methods requiring some knowledge and devotion, we will fail to save patients who die due to cardiovascular disease and infection, despite proper conduction of renal replacement therapy.

Many factors influencing the risk of sarcopenia development have been evaluated in a number of studies. The fact is, however, that the arsenal for the prevention and treatment still remains relatively poor. Although we know a lot of hormonal and molecular mechanisms, in every day practice we can safely apply the method, which has been successfully used for many years in elderly people with or without kidney disease: a proper nutrition to ensure an adequate intake of calories and protein combined with skillfully matched and regular exercise may act both as prevention and treatment.

In our center, there is no specific program for patients on maintenance hemodialysis. We believe, that in terms of PEW, every patient should be treated individually depending on his physical possibilities and expected effects. Unfortunately, the national health system in many countries does not support such aspects of ESRD patients' treatment. It seems, though, that a fresh glance is required, as hemodialysis itself is not enough for rescuing and improving patient's life. There are some conditions, which are necessary for hemodialysis to become efficient and successful. Patient's nutrition and good physical condition are the most important. Preventing and treatment of PEW is undisputedly a way to keep the patient in a good form.

Therefore, in our center we encourage the patients to some aerobic physical activity, which should be an element of every ESRD patient's treatment. Progressive resistance training is possible for realization individually by the patient, as there is no appropriate equipment in the dialysis center.

Physical activity should be supported by a proper dietary protein intake. 1,5 g protein/kg/24 h should be a minimum in patients on maintenance dialysis. It is worth to consider even a higher dose of protein in patients on intensive exercise treatment. Simple methods like implementing more meat and eggs in the diet proved successful in the number of our patients, who started to lose their lean body weight. The very important feature of such a treatment is that it may be continued at home.

We have also got some experience with hormonal treatment. One could summarize our attitude, that we let the patients on hemodialysis do what is banned in competitive sports: we givethem erythropoiesis-stimulating agents (ESAs) and anabolic steroids [125].

Anabolic steroids proved to be efficient in some of our patients, especially in the group with ESA hypo-responsiveness and hemosyderosis. Although we find a growth hormone a valuable agent in PEW treatment, we have not used it in our centre yet.

Megestrol acetate proved to be efficient in people with decreased appetite—it is worth to notice, that we have not observed side effects described in the literature so far.

In our center, we have also implemented a program for patients with CKD in stage IV K/DOQI. In the number of them, oral supplementation of NaHCO_3_ was introduced, resulting in slower progression of the kidney disease into ESRD. We have also noticed that such treatment may lead to reduction of PEW symptoms in an early period after hemodialysis initiation.

## Figures and Tables

**Table 1:** Review of the most important randomized controlled trials concerning nutrition treatment in ESRD patients.

Author	Implemented treatment	*N*	Results
Sundell et al. [96]	Pro-Stat 64 administered during hemodialysis	6	(i) Increased essential, nonessential, and total plasma amino acids concentration. (ii) Whole-body protein breakdown and net protein balance became statistically significantly better during HD in a dose-dependent manner.
Allman et al. [97]	Polycose-glucose polymer	9	(i) A mean increase in body fat of 1.8 kg and the lean body mass increased by 1.3 kg. (ii) The weight gain maintained after 6 months.
Milano et al. [98]	Glucose polymer	27	(i) Increase in body weight, body mass index, triceps skinfold, and brachial circumference at the end of the third month. (ii) Results were confirmed at 6 months in 18 patients that completed the study (mean body weight gain—2.4 kg). (iii) The nutritional status improved in only 4 patients at the end of the study.
Kuhlmann et al. [99]	Dietary treatment—3 groups: A: 45 kcal/kg/d and 1.5 g protein/kg/d; B: 35 kcal/kg/d and 1.2 g protein/kg/d; C: spontaneous intake supplemented with 10% of mean protein and energy intake	18	(i) Weight gain (1.2 ± 0.4 kg) observed only in group A. (ii) Serum albumin levels increased by 1.0 ± 0.5 g/L only in group A.
Patel et al. [100]	Dietary supplements	17	(i) Dietary supplements significantly increased both the nPCR and the total protein intake at 2 months and after 8 months. (ii) No change in the nutritional status of the subjects.
Hiroshige et al. [101]	Oral branched-chain amino acids (BCAAs) supplementation (12 g/day)	28	(i) Anorexia and poor oral protein and caloric intakes improved. (ii) The improvement in plasma BCAA levels. (iii) Increase and mean plasma albumin concentration.
Leon et al. [102]	Identification and intervention on nutritional barriers (depression, poor knowledge, poor appetite, help with shopping or cooking, low fluid intake, inadequate dialysis dose, depression, difficulty chewing, difficulty swallowing, gastrointestinal symptoms, and acidosis)	180	(i) Intervention patients had greater increases in albumin levels compared with control patients after 1 year. (ii) Greater increases in energy intake and protein intake in the intervention patients. (iii) The intervention most effective for barriers related to poor nutritional knowledge, help needed with shopping or cooking, and difficulty swallowing.
Fouque et al. [103]	Renilon 7.5(R) daily for 3 months	86	(i) Increased DPI and DEI compared to control group. (ii) No difference in serum albumin and prealbumin changes between groups. (iii) Improved SGA and QOL.
Caglar et al. [104]	Oral nutritional supplement specifically formulated for CHD patients	85	(i) Significant increases in concentrations of serum albumin and serum prealbumin. (ii) SGA score increased 14% by the end of the study.
